# Ribbing disease

**DOI:** 10.4103/0971-3026.59754

**Published:** 2010-02

**Authors:** Philson J Mukkada, Teenu Franklin, Rangasami Rajeswaran, Santhosh Joseph

**Affiliations:** Department of Radiology and Imaging Sciences, Sri Ramachandra Medical College and Research Institute, Sri Ramachandra University, Chennai - 600 116, India

**Keywords:** Ribbing disease, imaging, sclerosing

## Abstract

Ribbing disease is a rare sclerosing dysplasia that involves long tubular bones, especially the tibia and femur. It occurs after puberty and is reported to be more common in women. In this article we describe how Ribbing disease can be differentiated from diseases like Engelmann-Camurati disease, van Buchem disease, Erdheim-Chester disease, osteoid osteoma, chronic osteomyelitis, stress fracture, etc.

## Introduction

Ribbing disease is a rare sclerosing dysplasia involving the long tubular bones, especially the tibia and femur. In order to avoid unnecessary treatment, it is important to differentiate this entity from other sclerosing dysplasia like Engelmann-Camurati disease, Van Buchem disease, Erdheim-Chester disease, osteoid osteoma, chronic osteomyelitis, stress fracture and rarely, neoplasms.[1] There are very few case reports, so far, which have described the findings in all the imaging modalities in a single patient.[2,3] We would like to report a case of Ribbing disease, with emphasis on how it can be differentiated from its close mimics.

## Case Report

A 37-year-old woman presented with pain in the right leg of six months' duration and pain in the left leg of four weeks' duration. While the pain in the left leg was mild, that in the right leg had increased progressively. Based on the radiographic features, she was treated with antibiotics elsewhere as a case of low-grade osteomyelitis and was referred to our institution for further management as there was no improvement in the symptoms. There was no history of similar illness in her family. Her general examination was unremarkable. Local examination did not reveal any significant findings except for mild tenderness. Her routine blood investigations were unremarkable.

**Figure 1:**
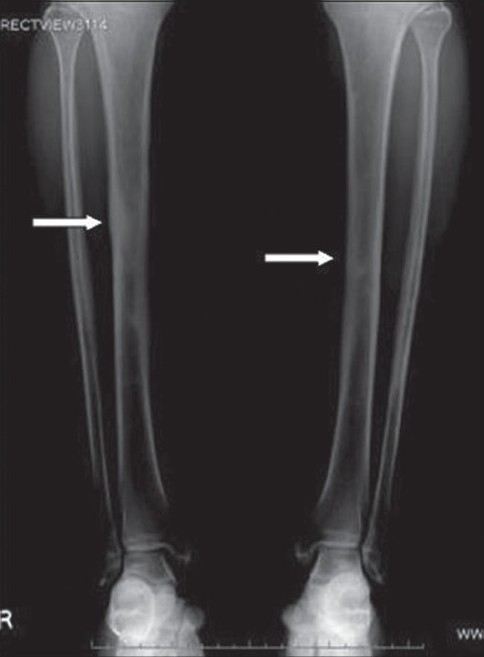
Frontal radiograph of both legs reveals sclerosis of the mid diaphysis of both tibias, right more than left (arrows)

**Figure 2:**
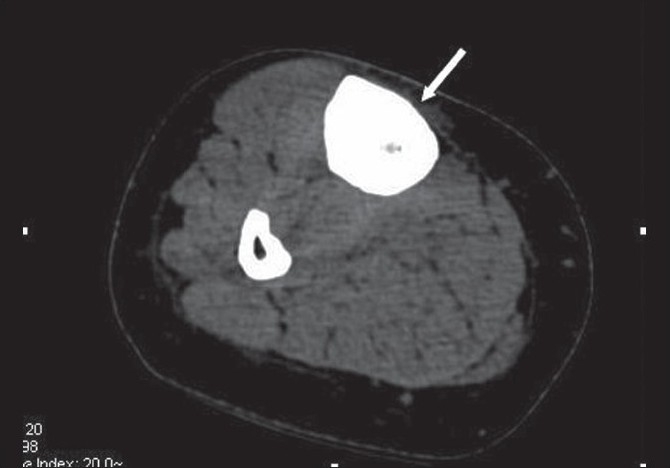
Axial plain CT scan of the right tibia demonstrates severe cortical thickening, with almost complete obliteration of the medullary cavity (arrows)

**Figure 3 (a,b):**
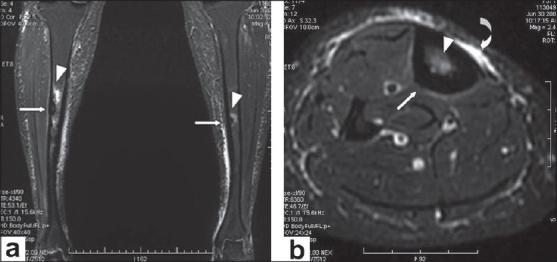
Short tau inversion recovery coronal (a) and axial (b) images show cortical thickening (arrows) with bone marrow edema in the diaphysis of both tibias (arrow heads) and minimal adjacent soft tissue edema

**Figure 4 (a,b):**
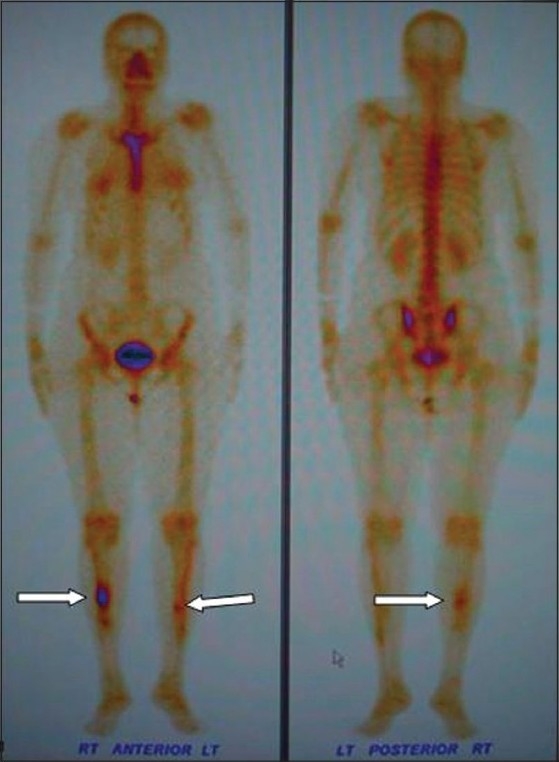
Anterior (a) and posterior (b) projections of a whole body Tc-99 m methylene diphosphonate bone scan show focal intense activity (arrows) in both tibial diaphyses (right more than left)

A frontal radiograph of both legs demonstrated sclerosis with cortical thickening involving the diaphyses of both tibias, which was more pronounced on the right side [Figure 1]. A CT scan showed circumferential cortical thickening with almost complete replacement of the medullary cavity [Figure 2]. MRI revealed similar findings; in addition, it revealed bone marrow edema and minimal adjacent soft tissue inflammation [Figure 3a and b]. Whole body Tc-99 m methylene diphosphonate (MDP) bone scan showed focal areas of increased tracer uptake in both tibial diaphyses, which were more on the right side [Figure 4]. No focus of increased tracer uptake was seen elsewhere in the skeleton. Biopsy from the right tibial diaphysis showed cortical thickening with packed trabeculae, suggestive of Ribbing disease.

## Discussion

Ribbing disease is a nonhereditary sclerosing dysplasia of unknown etiology affecting the diaphyses of tubular bones with sparing of epiphyses; it especially affects the tibia and femur. It was first described in 1949 by Ribbing.[4] It is a disease of adults and the incidence is reported to be more in women,[4] our patient also being a woman in her thirties. The typical presenting symptom is pain, which may be progressive in nature and associated with physical activity. Usually a self-limiting disorder, it may progress in some patients.

Radiographically, it is characterized by cortical and medullary sclerosis confined to the diaphysis, with sparing of the epiphysis. CT and MRI can delineate the extent of disease, reveal asymmetricity if there is bilateral disease, and also help in ruling out any associated cortical break, periosteal reaction, or soft tissue component. These findings help in differentiating Ribbing disease from other conditions like osteosarcoma, lymphoma, and metastasis. MRI shows low-signal-intensity areas in the medullary cavity, corresponding to the sclerosis, on all pulse sequences. Associated increased signal on short tau inversion recovery (STIR) and proton-density fat-saturation sequences may be seen; this represents bone marrow edema, which is believed to be the cause of pain in these patients.[5] Cross-sectional imaging also helps in differentiating Ribbing disease from osteoid osteoma, where the characteristic nidus can be seen. Bone scintigraphy shows increased uptake of Tc-99 MDP in the involved diaphysis and helps in ruling out abnormal tracer concentration elsewhere in the skeleton.[6]

The close mimic of Ribbing disease is Engelmann-Camurati disease, a hereditary sclerosing dysplasia with autosomal dominant inheritance that affects children. Both diseases involve the diaphyses of long tubular bones with sparing of the epiphyses, but Engelmann-Camurati disease is bilaterally symmetrical, whereas Ribbing disease is either unilateral or, if bilateral, always asymmetric. The presence of skull bone involvement, gait and neurologic abnormalities, and anemia with extramedullary hemopoiesis favors Engelmann-Camurati over Ribbing disease.[1,6,7] Van Buchem disease (hyperostosis corticalis generalisata familiaris) is a hereditary sclerosing dysplasia that affects the long bones; the diaphysis is predominantly affected but the epiphysis can also be involved. There is conspicuous involvement of the jaws.[8] Other sclerosing dysplasia like melorheostosis and osteopetrosis can be differentiated from Ribbing disease by their characteristic pattern. Bilateral involvement of tubular bones in Ribbing disease helps in excluding chronic sclerosing osteomyelitis, which is usually unilateral.

Erdheim-Chester disease is a systemic histiocytic disorder with sclerosis of long bones, but the clinical features and the involvement of bone epiphyses as well as of other systems/tissues like pericardium, lungs, and retroperitoneum helps in ruling out this rare disorder.[9] Stress reaction can mimic Ribbing disease radiologically, but the history and the relatively discrete involvement seen in the former may help in diagnosis. Neoplasms like lymphoma, metastasis, and multicentric osteosarcoma can easily be differentiated from Ribbing disease by the clinical history and imaging.

## Conclusion

Ribbing disease is mainly a diagnosis of exclusion. The important diagnostic features of Ribbing disease are adult onset, female predominance, absence of similar disease in family members, pain, normal serum markers; sclerosis of the diaphyses of long tubular bones, with sparing of epiphyses (especially affecting the tibia); the bilateral asymmetric involvement of bone, without any cortical break, periosteal reaction, and soft tissue component; and the absence of involvement of the skull base, the jaw, or other systems.
